# Whole-Body Disposition and Metabolism of [^14^C]-2,4,4′-Trichlorobiphenyl (PCB28) Following Lung Administration in Rats

**DOI:** 10.1021/acs.est.5c07982

**Published:** 2025-10-23

**Authors:** Yau Adamu, Nicole Marie Brandon, Andrea Adamcakova-Dodd, Hui Wang, Peter S. Thorne

**Affiliations:** † Human Toxicology Program, 4083 The University of Iowa, Iowa City, Iowa 52242, United States; ‡ Department of Occupational and Environmental Health, The University of Iowa, Iowa City, Iowa 52242, United States

**Keywords:** polychlorinated biphenyl, PCB28, forever chemicals, toxicokinetics, ADME, pulmonary

## Abstract

Toxicities of lower-chlorinated biphenyls (LC-PCBs) have drawn increasing attention due to growing evidence of their presence in school indoor air, with 2,4,4′-trichlorobiphenyl (PCB28) being a prevalent congener. The tissue levels and characteristics of individual PCB congeners determine the PCB toxicity. Yet, the tissue disposition time course of individual LC-PCBs following pulmonary exposure is largely unknown. To address this data gap, we investigated the whole-body disposition, metabolism, and elimination kinetics of PCB28 in rats following intratracheal lung dosing of radiolabeled [^14^C]-PCB28. Blood, 36 tissue types, 6 digestive matters, and excreta were sampled at specific intervals (12, 25, 50, 100, 200, 400, 720, and 1440 min) postexposure. The pulmonary uptake of PCB28 was nearly complete at 99.9 ± 3.5%. PCB28 rapidly distributed across multiple tissues, initially accumulating in lung and liver, followed by redistribution to the adipose and skin. PCB28 exhibited linear toxicokinetics (TK) in highly perfused tissues, resulting in dose-proportional increases in the area under the concentration–time curves. In contrast, adipose tissue, skin, and prostate displayed nonlinear TK. The elimination half-life of [^14^C]-PCB28 was 12 h, with elimination primarily via the fecal route. This study provides new data on the toxicokinetics of PCB28 following pulmonary exposure to support the development of TK models for evaluating the health risks posed by this persistent environmental pollutant.

## Graphical abstract

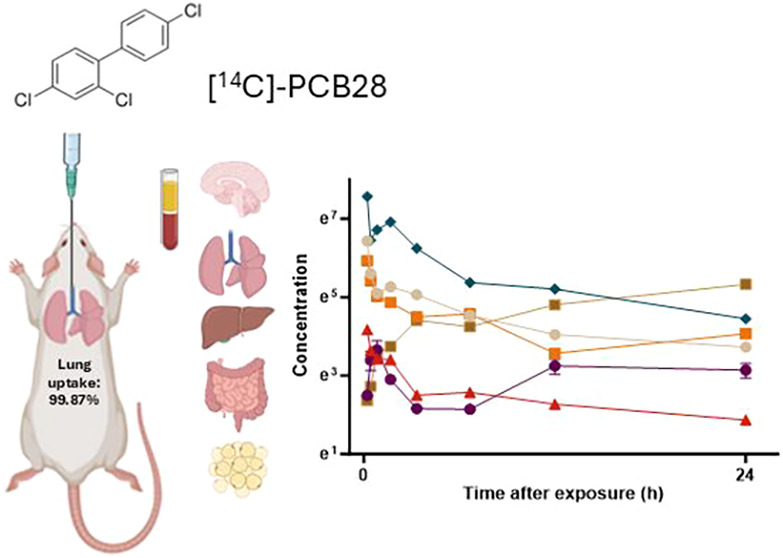

## 1 Introduction

Polychlorinated biphenyls (PCBs) were extensively employed in industrial applications from the late 1920s until their production ban in 1979 due to their detrimental impacts on human health and the environment.^1,2^ Despite the 46-year ban on their intentional production, these compounds continue to be widely distributed in the environment, as evidenced by multiple studies.^3–9^ The period of extensive school construction (1950–1980) coincided with peak PCB utilization in building materials, raising concerns for the health of vulnerable populations, especially schoolchildren and teachers, as well as pregnant women and children residing in older buildings with similar construction materials. Indoor air generally contains higher PCB concentrations than outdoor air^4,10–13^ and early-life exposures contribute to cumulative impacts. Furthermore, general populations living near industrial sites or in areas with high ambient PCB levels are also at elevated risk as PCB28 is one of the most abundant congeners in indoor and ambient air. Despite substantial declines in dietary PCB levels, atmospheric PCB concentrations remain relatively abundant. While dietary intake is still the major PCB exposure pathway for many members of the general population, evidence is mounting that inhalation exposure to PCBs now surpasses dietary exposure in some situations.^4,14–17^ However, insufficient data exist regarding the toxicokinetic profiles of individual PCB congeners following inhalation, hindering risk assessment.^18–20^ Prior research explored the fate of inhaled lower-chlorinated PCBs (LC-PCBs), including dichlorinated PCB11 and tetra-chlorinated PCB52 in animals, revealing a high pulmonary absorption, rapid distribution, extensive biotransformation, and excretion through urine and feces.^21–23^ Investigation of structure–activity relationships has shown that PCB congeners differ markedly in their patterns of cytochrome P450 (CYP) induction, influenced by the position and number of ortho-chlorine substituents on the biphenyl structure.^24–27^ Non-ortho-substituted PCB congeners are often assumed to exhibit analogous toxicokinetic behavior due to their shared lack of ortho-chlorine substitutions. However, this assumption oversimplifies the complex interplay between structural features and biological activity. For example, PCB congeners with a non-ortho-substituent, such as PCB11, PCB3, and PCB77, might be anticipated to exhibit analogous toxicokinetic behavior. However, while PCB11 and PCB3 are non-dioxin-like, low-chlorinated congeners that undergo rapid metabolism and elimination, PCB77 is a dioxin-like congener with a coplanar structure that facilitates aryl hydrocarbon receptor (AhR) binding. This results in markedly slower metabolism and prolonged tissue retention and bioaccumulation. These distinctions highlight that ortho-substitution alone is insufficient to predict toxicokinetic behavior and congener-specific evaluations are essential. Furthermore, uncertainties persist regarding other LC-PCB congeners with one or more ortho-substituents, which are abundant in the air. The 2,4,4′-trichlorobiphenyl (PCB28) congener has been detected across diverse environmental matrices such as food, water, sediment, and urban and indoor air due to its physicochemical attributes.^28–32^ Air measurements within school environments have shown PCB28 concentrations up to 1689 ng/m^3^,^10,11,28,31^ making the inhalation route of exposure a significant contributor to total PCB exposure. Exposure to PCB28 poses notable health risks due to its neurotoxicity, hepatotoxicity, and neurobehavioral effects.^33–35^ PCB28 and metabolites are frequently detected in human tissues.^4,14,36–38^ The existing toxicokinetic studies of PCBs have focused on oral and intravenous high-dose exposures to PCB mixtures and highly chlorinated (HC)-PCBs under prolonged exposure conditions.^39–44^ However, considering that PCBs are typically encountered at low levels in real-world scenarios, fundamental insights into the acute tissue distribution, metabolic processes, and elimination of individual PCB congeners are essential for assessing risks associated with intermittent pulse-like inhalation exposure. Particularly, the behaviors of LC-PCBs in the pulmonary context may differ from those observed with oral or intravenous routes. Parenteral (e.g., intraperitoneal, intramuscular, subcutaneous) and oral (e.g., gavage) are major routes of administration commonly utilized in toxicokinetic studies.^20,45–49^ These methods were selected for their ease of use and precise dose control.^20,50,51^ Although inhalation exposure represents real-world exposures to airborne LC-PCBs, it is less amenable to precise measurement of PCB52 deposited in the lung or exhaled into the air, which are important kinetic parameters for conducting human risk assessment. Therefore, we used intratracheal instillation to directly expose lungs to PCB28, allowing for the precise quantification of loss and efficient use of the limited amount of ^14^C-PCB28. This approach improved the translational impact of the study by targeting the lungs and associated tissues, resulting in adverse biological responses.^9,17,21,52^ Inhaled toxicants following intratracheal and direct inhalation are readily available to the lungs and systemic circulation via translocation through pulmonary epithelium or retention in the lung.^52,53^ In this study, we first performed *in silico* predictions of PCB28 metabolites and then conducted *in vivo* toxicokinetics and metabolism studies of [^14^C]-labeled PCB28 ([^14^C]-PCB28) dosed to the lungs to elucidate pulmonary uptake, distribution, metabolism, and elimination across 36 tissues, digestive segments, and excreta.

## 2 Materials and Methods

### 2.1 In Silico Prediction of PCB28 Metabolites Using ADMET Predictor

In this study, we employed the ADMET Predictor version 12 (Simulations Plus, Lancaster, CA) to conduct *in silico* predictions of metabolites for PCB28. The chemical structure of PCB28 was represented in Molfile format utilizing ChemBioDraw Ultra (PerkinElmer, Inc., Cambridge, MA), subsequently uploaded into ADMET Predictor, as previously documented.^54–56^ Through the utilization of default settings within the Metabolism Module of ADMET Predictor, the assessment of potential metabolite structures for PCB28 was performed to aid the preliminary understanding of the fate of PCB28, facilitating the interpretation of its metabolic pathways in rats.

### 2.2 Chemicals

Radiolabeled PCB28, [phenyl-^14^C­(U)] (PCB28), with a specific activity of 60.0 mCi/mmol and with both chemical (HPLC, UV250) and radiochemical (HPLC) purity exceeding 99%, was procured (ViTrax, Placentia, CA). The radioactive ^14^C isotope was uniformly distributed within the biphenyl moiety. All nonradiolabeled PCBs, along with their corresponding metabolites, employed in this investigation were synthesized, validated, and supplied by the Synthesis Core of the Iowa Superfund Research Program (ISRP). The synthetic procedures and characterizations were previously described.^57–59^ Aqueous-based solubilizer (Solvable, catalog no. 6NE9100) and scintillation cocktail (ULTIMA-GOLD, catalog no. 6013329), both from PerkinElmer (Waltham, MA) were used for tissue digestion and liquid scintillation counting, respectively. The PCB surrogate standard, 2,4,4′-trichlorobiphenyl (PCB28) was synthesized and authenticated as described.^57–60^ Other standards 2,2′,3,4,4′,5,6,6′-octachlorobiphenyl (PCB204; internal standard), 2,2,3′,4-trichloro-4′-hydroxybiphenyl (4′-OH-PCB25; surrogate standard), 2,3′,4′-trichlorobiphenyl-4-ol (4′-OH-PCB 33; surrogate standard), and 2,2′,4′,5-tetrachloro-4-MeSO_2_-biphenyl (4-MeSO_2_-PCB49) were purchased from AccuStandard (New Haven, CT, U.S.A.).

### 2.3 Preparation of Exposure Formulation

The radiolabeled PCB28, a solid with an initial activity of 0.5 mCi, was dissolved in 100 μL of hexane and stored at −20 °C as a stock solution. The dosing solution was prepared by adding 10 μL of hexane to 10 μL of the stock solution. After Tween 80 was incorporated into saline (0.9% sodium chloride) solution as a surfactant, Tween 80 in saline was added in five aliquots, sonicating between each addition to form an emulsion. The final [^14^C]-PCB28 dosing solution consisted of 2% hexane and 0.1% Tween 80 in saline (referred to as a vehicle). Fresh 1 mL quantities of the dosing solution were periodically made, and a portion was analyzed for radioactivity using a liquid scintillation counter (Beckman LS 6500, Fullerton, CA) to ensure that a consistent dose of PCB28 was administered to each animal.

### 2.4 Animal and Exposure

Male Sprague–Dawley rats (8 weeks old) weighing 230–285 g (mean of 256 g) were used in this study (Envigo, Indianapolis, IN). Male rats were selected to maintain consistency with previous toxicokinetic investigations of lower-chlorinated PCBs, specifically the study on intratracheally instilled ^14^C-labeled dichlorinated PCB11,^21^ which also utilized male rats. This approach facilitated direct comparison across studies and contributed to a more comprehensive toxicokinetic data set for LC-PCBs following inhalation exposure. A PCB toxicokinetic study being published separately investigated placental transfer and fetal uptake in pregnant females. All animals were handled in compliance with an approved Institutional Animal Care and Use Committee protocol (protocol#: 3091097 approved on 10/12/2023). Rats were pair-housed in a light and temperature-controlled environment with access to food and water *ad libitum*. After 1 week of acclimatization, rats were assigned to either a high-dose (HD) group (*n* = 9) or a low-dose (LD) group (*n* = 5) at random. Control rats (*n* = 3) were exposed to a single dose of unlabeled PCB28 (42 μg in 200 μL) and necropsied 50 min after exposure to establish background levels for radioactivity calculations in blank tissues. The sample size of the study was based on previous studies of toxicokinetics and mass balance.^21,61^ Findings from those studies demonstrated that three rats per time point for plasma toxicokinetic study were more than sufficient to gain adequate absorption, distribution, metabolism, and excretion information while minimizing the number of animals required for the study and ensuring efficient use of the extraordinarily expensive [^14^C]-PCB28. Therefore, animal numbers were customized between groups to most efficiently utilize the limited available ^14^C-PCB28 stock in our lab.

Animals in the HD group were intratracheally (i.t.) administered 42 μg of a one-time single dose of [^14^C]-PCB28 in 200 μL of vehicle, while animals in the LD group received 4.2 μg of [^14^C]-PCB28 in 100 μL of vehicle. The i.t. route for dosing was selected due to the limited availability and extraordinary cost of the radiolabeled PCB28, and to avoid ^14^C contamination of the inhalation exposure apparatus. All i.t. instillation was performed under light anesthesia (4% isoflurane via a precision vaporizer Fortec, Cyprane, Keighley, UK). Rats recovered from the anesthesia within 2 min while held within a cylindrical glass chamber (2 L), equipped with drainage ports to facilitate urine collection and a metallic screen to segregate urine from feces (as illustrated in Figure S1). Air was drawn from the chamber at 6.0 L/min through a cartridge containing XAD-2 polymeric resin (Amberlite, Millipore Sigma, Burlington, MA) to capture any exhaled [^14^C]-PCB28. For the longer time points (720 and 1440 min), individual rats were placed in metabolic cages following a similar protocol for excreta and exhaled breath collection. After exposure, the inner walls of the chamber were rinsed separately with water, acetone, and hexane (25 – 45 mL) and wiped to measure radioactivity. Rat fur was also wiped and analyzed for radioactivity. The rats exposed to the HD were euthanized using isoflurane and exsanguination via cardiac puncture at 12, 25, 50, 100, 200, 400, 720, and 1440 min postexposure (*n* = 1 per time point except *n* = 2 for 200 min). Animals exposed to the LD were similarly necropsied at time points of 2, 12, 50, 200, and 720 min postexposure (*n* = 2). Euthanasia was consistently performed during the light cycle, except in the case of the 720 min time point, wherein euthanasia occurred outside the light cycle. Our prior study with [^14^C]-PCB11 demonstrated a high reproducibility of our replicated dosing.^21^

Serum and blood cells were separated. Two to six technical replicates were collected from 36 tissue types and digestive matter (diluted in ultrapure water) from 5 different GI tract locations. Muscle and adipose tissue samples were obtained from various locations. The collected tissue samples were placed in scintillation vials containing 1 mL of solvable cocktail and kept at 37 °C overnight for complete solubilization. To enable subsequent analyses, the solubilized tissue samples underwent a 2-h heating phase at 50 °C. For the bone, feces, and digestive matter, 1 mL of isopropyl alcohol was added. For samples displaying darker coloration, a controlled volume of 30% hydrogen peroxide (100 to 300 μL) was slowly added in increments to remove potential interference. After adding H_2_O_2_, the samples were heated to remove the O_2_. Subsequently, 10 mL of scintillation cocktail was added to each sample, and radioactivity was quantified using a liquid scintillation counter (Beckman Coulter LS 6500). The acetone, hexane, and water rinses from the metabolic chamber were analyzed to quantify the residual radioactivity associated with exhalation and excretion. To ensure accurate quantification, we used quench correction curves established through the utilization of a [^14^C]-toluene standard to ensure activity counting efficiency and accurate quantification (PerkinElmer, Waltham, MA).

### 2.5 Extraction and Separation of Parent PCB28 and Metabolites

Extractions of parent PCB28 and its hydroxylated, methyl sulfone, and conjugated metabolites in serum, liver, adipose, and kidney samples were conducted using our previous protocol.^21^ Individual extractable metabolites were separated using a series of liquid–liquid extractions, employing a graded array of solvents with increasing polarity to facilitate the partitioning of the analytes according to their inherent lipophilic characteristics (Figure S2). Subsequently, individual fractions were classified into respective categories, including the native PCB moiety, hydroxylated PCB (OH-PCB), methyl sulfonyl-PCB (MeSO_2_-PCB), conjugated metabolites, and unextractable components. Analytical recoveries were quantified for each extraction and separation step. Tissue samples (200–400 mg wet weight) were prepared for analysis in a two-step homogenization process using 2 mL of hexane/acetone solvent (50:50, vol/vol). The resultant solution was centrifuged, and then the pellet underwent two successive extractions with 1.5 mL of dichloromethane (DCM), chloroform/methanol (50:50 vol/vol), methanol/water (50:50 vol/vol), and water, respectively. The supernatant from the DCM extraction was shown to contain hydroxylated metabolites, whereas the top fractions from the methanol/water extraction had conjugated PCB metabolites. The resultant supernatant from the hexane/acetone extraction was rinsed with Milli-Q water, extracted with a 0.5 M potassium hydroxide (KOH) solution in 50% ethanol, and triple rinsed with 400 μL of hexane. The hydrophobic fraction was further purified by rinsing with 1 M hydrochloric acid (400 μL). Simultaneously, a plausible MeSO_2_-PCBs fraction was extracted with 500 μL of anhydrous dimethyl sulfoxide (DMSO), followed by triple hexane rinses (3 × 400 μL). The residual hydrophobic phase was quantified as native PCB28. The quantification of unlabeled PCB28, OH-PCBs (as MeO-PCB derivatives), and MeSO_2_–PCBs in standards spiked samples was performed on a gas chromatograph (Agilent 6890N) equipped with a ^63^Ni μ-ECD detector and SLB-5MS capillary column (60 m × 0.25 mm I.D., 0.25 μm film thickness; Supelco, St Louis, MO). Subsequently, individual separated fractionation from [^14^C]-PCB28-exposed tissue samples were transferred to a prepared scintillation vial, followed by the addition of 10 mL cocktail solution and analysis using the scintillation counter as described above. Any quantifiable radioactivity in the residual pellet was treated as unextracted PCB compounds.

### 2.6 Data Analysis

Data were subjected to descriptive analysis of the total [^14^C]-PCB28 concentration (disintegration counts per minute [dpm]/mg of wet weight) of individual tissue types, followed by plotting log of concentration versus time data of multiple organs (Prism 10.2.0, GraphPad Software, Boston, MA). The total radioactivity of each organ was calculated as the sum of radioactivity per mass found in each sample multiplied by the organ weight. For the digestive matter and feces, radioactivity was taken as the product of the sample concentration and total wet weight divided by total dry weight.^21^ Total weights for adipose tissue, skin, muscle, bone, and blood were determined based on documented percentage body weights of 6, 16, 44, 7, and 6%, respectively.^62^ The exhaled amount of radioactivity comprised the amount in the XAD-2 cell cartridge, the chamber rinses and wipes, and the rat wipes. The degree of pulmonary absorption was calculated by subtracting exhalation and feces excretion data from 100% of the nominal dose administered to each rat and calculating the mean percentage and apparent absorption efficiency.^63^

The toxicokinetic parameters were estimated using Phoenix Pharmacokinetic and Pharmacodynamic (PK/PD) Platform Version 8.4.^64–66^ Briefly, a noncompartmental pharmacokinetic analysis (NCA) was conducted on the plasma concentration–time pooled data resulting from the administration of [^14^C]-PCB28 via the intratracheal route. The important parameters of interest were derived from the plasma concentration–time profiles for each experimental group. Specifically, the maximal concentration (C_max_) of PCB28 in plasma and the corresponding time point (T_max_) of its attainment were also determined. The elimination rate constant (Ke) was estimated by fitting the terminal log–linear segment of the plasma concentration–time curve, employing a least-squares approach. The area under the plasma concentration–time curve within the interval of 0 to 24 h (AUC_0–24 h_) was computed employing the linear trapezoidal rule. Correspondingly, the area under the curve spanning from time zero to infinity (AUC_0‑∞_) was extrapolated to infinity using the equation AUC_0–24 h_ + C_24 h_/Ke, with C_24 h_ signifying the plasma concentration measured at 24 h. The dose-normalized total area under the curve (AUC_0‑∞_/dose) was computed to account for potential dose variations. The terminal elimination half-life (*T*_1/2_) was estimated by using the apparent terminal rate constant. The partition coefficient (P_tissue/blood_) of PCB28 in different tissues was calculated using the area method,^67^ by taking the ratios of the AUC_0‑∞_tissue_ in each tissue to the AUC_0‑∞_blood_. The absorption efficiency (apparent bioavailability) was estimated as previously described.^63,68^ The mean AUC between metabolites was compared using the Kruskal–Wallis test due to violation of the parametric assumptions. Post hoc analysis and multiple pairwise-comparison tests were performed using the Dunn test, where two metabolites were significantly different from each other. *P*-value ≤ 0.05 was considered statistically significant.

## 3 Results and Discussion

### 3.1 Predicted Metabolites and Metabolizing Enzymes

*In silico* metabolite predictions demonstrate PCB28 as a substrate for specific cytochrome P450 isoforms including CYP1A2, CYP2A6, CYP2B6, CYP2C19, CYP2C8, CYP2C9, CYP2E1, and CYP3A4 (Figure 1). Other enzymes involved include the UDP-glucuronosyltransferase isoforms: UGT1A1, UGT1A3, UGT1A6, UGT1A8, UGT1A9, UGT1A10, UGT2B7, and GT2B15. Although the software was not equipped to predict the metabolic pathway involved in the formation of sulfate conjugates catalyzed by the sulfotransferase enzymes (SULTs), sulfate metabolites of PCB28 have been identified in animal and human samples.^29^ The primary metabolites include 2′-OH-PCB28; 3′-, 5-, and 6-glucuronide-PCB28; 3-sulfate-PCB28; methyl sulfone, and quinone PCB metabolites. Our findings demonstrate strong agreement with experimental data. Several predicted hydroxylated metabolites of PCB28, such as 2′-OH-PCB28, 3-OH-PCB28, 3′-OH-PCB28, 4′-OH-PCB31, 4-OH-PCB25, 5-OH-PCB28, and 5-OH-PCB28, have been identified in both human plasma, rat and mouse tissues, and flies following PCB28 exposure.^29,36,42,45,69–74^ The 3-sulfate-PCB28, 4-sulfate-PCB25, and methyl sulfone metabolites have been experimentally detected.^42,73,75–77^ These metabolites were confirmed or detected using analytical techniques including liquid chromatography–tandem mass spectrometry (LC-MS/MS) and gas chromatography–mass spectrometry (GC-MS), with synthetic standards aiding structural validation in rat, mice, flies, human hepatic microsomes; recombinant SULT assays, and LSC for radiolabeled kinetic studies.

**Figure 1:**
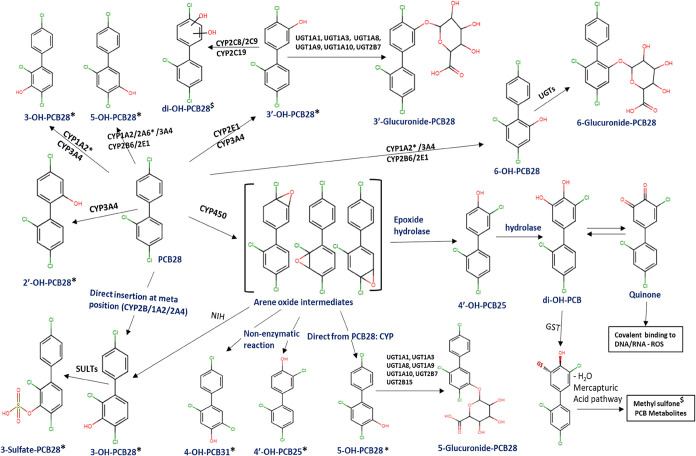
Predicted metabolites and enzyme substrates and proposed metabolic pathways for PCB28. Metabolites labeled with an asterisk (*) after the name signify experimentally confirmed metabolites, while those with a dollar ($) sign are either detected metabolites using nontargeted or LSC analysis.

### 3.2 Efficiency of [^14^C]-PCB28 Delivery

Exhaled breath contributed a minuscule amount of the total recovered radioactivity (0.07 ± 0.04%), indicating that most of the administered dose was rapidly taken up by the lung. The nominal recovered radioactivity of the administered dose accounted for in the collected tissues that were analyzed was 83 ± 5% for the HD and 74 ± 13% for the LD. The recovery percentage data across tissue samples was normalized to 100%. Welch’s test (which accounts for unequal variances) identified no significant difference in tissue radioactivity recovery between the HD and LD groups (*p* = 0.20). By the end of the 24 h study period, no more than 4% of the total radioactivity was excreted in the feces, leaving 88–92% of the total radioactivity within the body, suggesting an apparent bioavailability of up to 96% (Figure 2).

**Figure 2:**
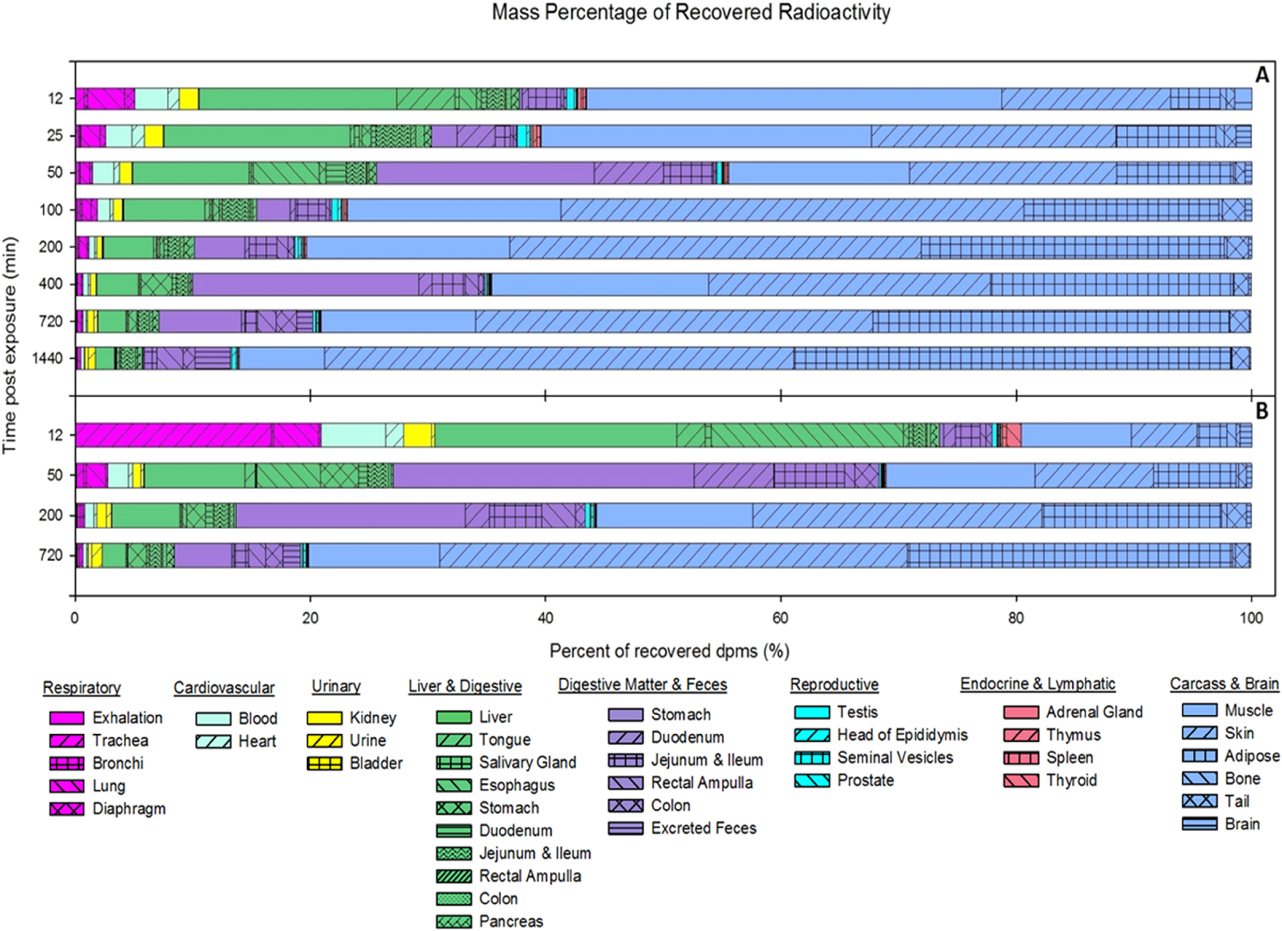
Mass percentage of recovered activity in each organ, tissue, and body compartment following lung dosing to [^14^C]-PCB28 at 42 μg (high dose) (A) and 4.2 μg (low dose) (B). The recovery for each time point was normalized to 100%.

### 3.3 Time-Course Tissue Distribution of [^14^C]-PCB28

The distribution of [^14^C]-PCB28 to 36 tissues, feces, and six different segments of digestive tissue and their respective content matter followed similar trends for both the HD and LD groups (Figure 2). The unlabeled PCB28-exposed tissue data from the control group were used as background levels (subtracted from the activity counts from the exposed tissues). Radioactivity was detected in every tissue 12 min after i.t. administration of PCB28. The radioactivity decreased between 12 and 50 min postexposure in many tissues, especially in the respiratory and cardiovascular systems. The quantified radioactivity reduced by 56–73% in the lung, 37–67% in the blood, 36–69% in the kidney, and 41–58% in the liver between these time points. Higher [^14^C] radioactivity was recovered from muscle, skin, and well-perfused tissues at early time points postexposure (lung, bronchi, liver, kidneys, esophagus, and heart), including tissues with a low proportion of whole-body activity, such as bronchi, seminal vesicles, testis, and diaphragm (suggesting higher loading), followed by redistribution to adipose and adipose-containing tissues, including skin and muscle. By the 24 h exposure time point, 88–92% of the radioactivity remained within the body, with the skin and adipose tissue sequestering 67–77% of the retained radioactivity. Fecal excretion was minimal, contributing no more than 4% to the total radioactivity at any given time point throughout the study period. Although the spatiotemporal distribution of [^14^C]-PCB28 and its metabolites throughout the tissues followed similar trends for both HD (Figure 3) and LD exposures (Figure 4), a few exceptions were noticed. At 12 min postexposure, approximately half (52.1%) of the remaining radioactivity was found in the muscle (35.3%) and liver (16.8%) in HD animals, while most was found in the liver (20.6%) and pulmonary regions (16.7%) in the LD group.

**Figure 3:**
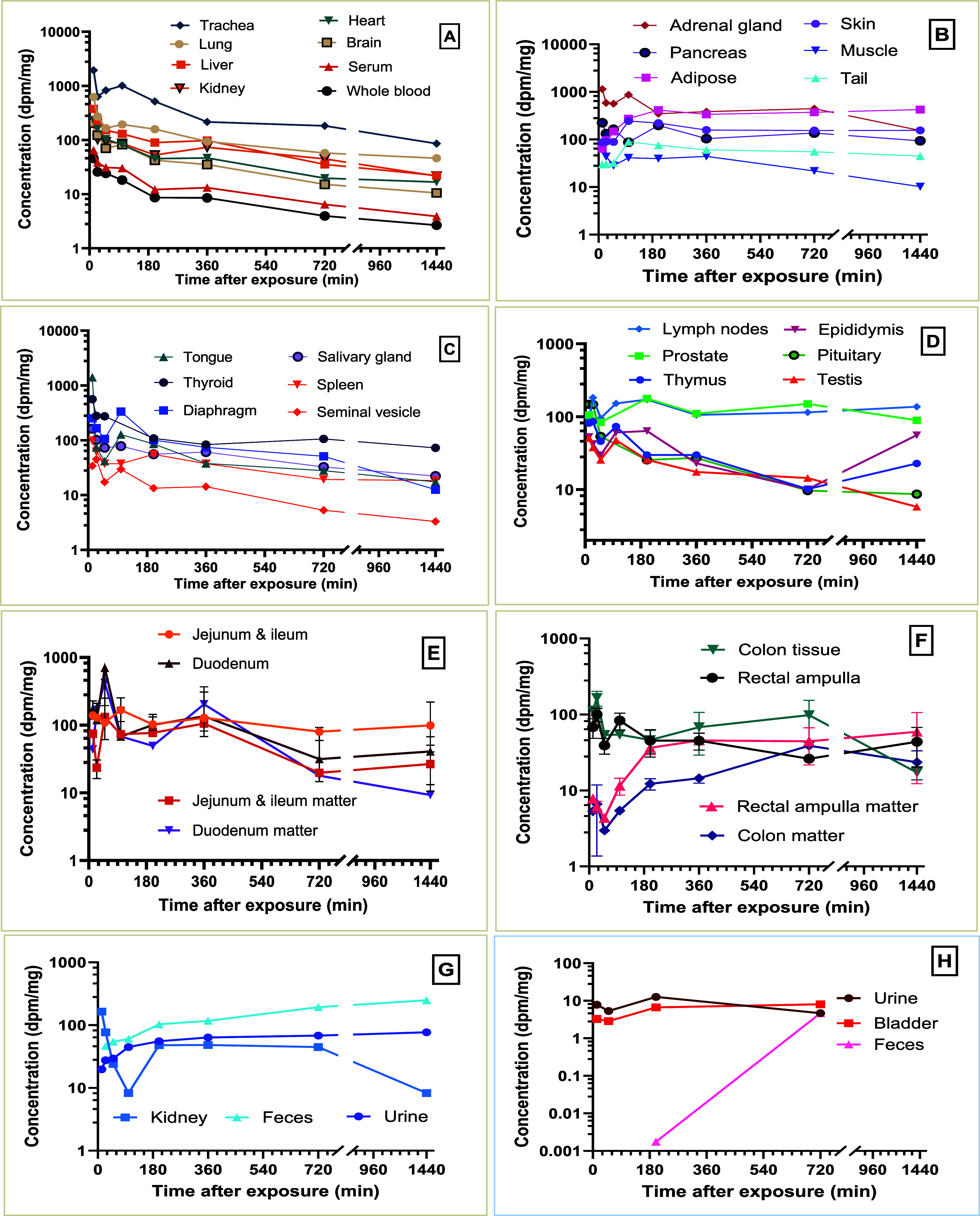
Time course of [^14^C]-PCB28 concentration change in high-dose tissues (A–G), demonstrating toxicokinetic profiles of PCB28 in the respiratory, cardiovascular, endocrine, and reproductive systems (A–D), adipose skin, muscle, tail, and adrenal glands (B), jejunum and ileum, and duodenum and their digestive matter (E), colon and rectal ampulla with their digestive matter (F), and in the kidney, urine, and feces (G). (H) Time course data for the urine, bladder, and feces from the low-dose treatment.

**Figure 4:**
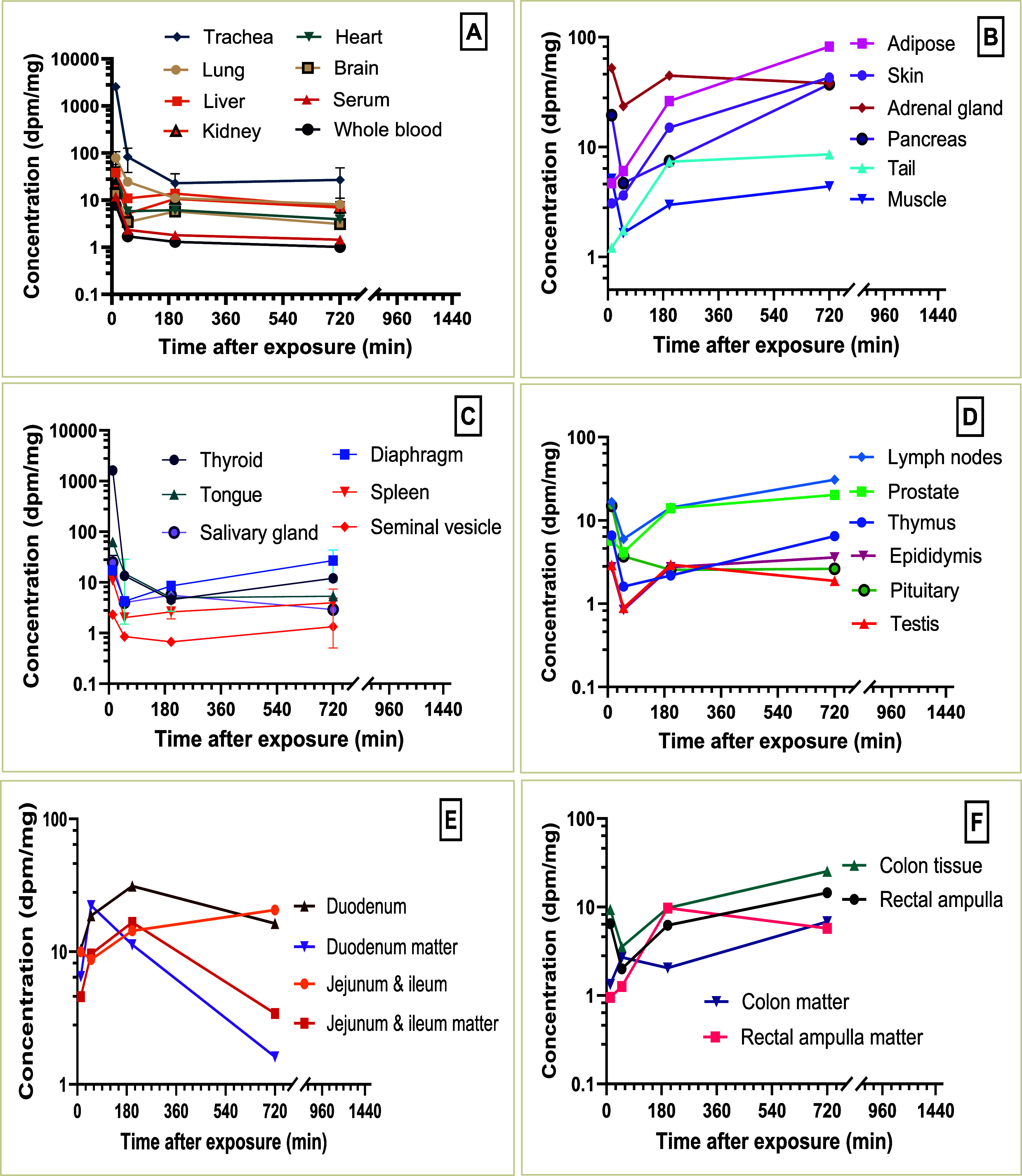
Time course of total [^14^C]-PCB28 concentration change in low dose tissues (A–F) demonstrating profiles of PCB28 in the respiratory, cardiovascular, endocrine, and reproductive systems (A, C, D), adipose skin, muscle, pancreas, tail and adrenal glands (B), in the jejunum and ileum, and duodenum tissues and corresponding digestive matter (E), in the colon and rectal ampulla tissues with their digestive matter (F) after pulmonary exposure.

The transient elevated radioactivity measured in the trachea and bronchial regions of the LD tissues indicated a slower tissue distribution than the HD tissues. A similar observation was recorded in the serum at the LD, which retained 92% more radioactivity than the HD for the same point, after which it declined steadily (Figures 2, 3A, and 4A). Total radioactivity in the muscle increased steadily between 25 and 720 min for rats exposed to LD (Figure 4B). HD rats exhibited a steady concentration until 360 min and a slow decline afterward (Figure 3B).

### 3.4 Toxicokinetic Parameters of [^14^C]-PCB28

The toxicokinetic parameters derived by NCA are presented in Table 1. The time to reach the maximum serum concentration (T_max_) of 0.065 dpm/mL (65.0 dpm/mg) was 12 min (0.21 h). For the remaining tissue compartments, the T_max_ values in adipose, skin, and prostate were 200 min (3.33 h), 100 min (1.67 h), and 200 min (3.33 h), respectively (Tables S1 and S2). The last observed serum concentration at 1440 min (24 h) postexposure (C_last_) was 4 dpm/mg. The serum apparent terminal *T*_1/2_ was 11.7 h (702 min) (Table 1). It was not possible to calculate clearance and volume of distribution directly from the extravascular route of administration because the exact PCB28 concentrations that gain access to the systemic circulation were unknown.

**Table 1:** Mean Toxicokinetic Parameters of PCB28 Obtained by NCA after Intratracheal Administration of [^14^C]-PCB28 Formulations to Male Rats (*n* = 2–3 Per Time Point)

parameters	units	mean (*C–V*%)
*T*_1/2_	h	11.7 (19.6)
T_max_	h	0.21 (14.4)
C_max_	dpm/mg	65.0 (4.4)
C_24_	dpm/mg	4.0 (28.6)
AUC_0–24_	h*dpm/mg	243.1 (13.5)
AUC_0‑∞_	h*dpm/mg	310.8 (7.4)
Vz/F	mg	5.83 (14.2)
Cl/F	mg/h	0.35 (7.3)

AUC_0‑∞_, area under the concentration–time curve from zero up to ∞ calculated using AUC_0‑∞_ = AUC_0–24_ + C_last_/Ke from observed concentration and terminal half-life values. Cl/F, apparent total serum clearance of ^14^C-PCB28 estimated CL/F = Dose/AUC_0‑∞_; Vz/F, apparent volume of distribution during terminal phase for nonsteady-state after extravascular dosing from Vz/F = Dose__ex_/(AUC_0‑∞_*Ke).

However, the ratios of volume of distribution to bioavailability (Vz/F) and clearance to bioavailability (Cl/F) were estimated to give approximated volume of distribution and total clearance of [^14^C]-PCB28 following i.t. administration.^78,79^ The apparent total serum clearance (Cl/F) of [^14^C]-PCB28 after i.t. administration was 0.35 mg/h (0.00035 L/h), and the apparent volume of distribution during the terminal phase (Vz/F) after single-dose extravascular administration was 5.83 mg. The estimated Cl/F and Vz/F for other organs were relatively minor compared to the totals, and the estimated values are likely within the expected ranges. The apparent terminal elimination half-life from the central compartment (*T*_1/2_) using the terminal rate constant was 11.7 h (Tables 1, S1, and S2). High C_max_ was observed in the bronchial tissues (2830 dpm/mg), followed by nonbronchial lung areas, thyroid, adipose, liver, skin, and prostate tissues, while lower C_max_ was found in the reproductive organs, including testis and seminal vesicles (Tables S1 and S2). The highest C_last_ was recorded in the adipose, followed by skin, pancreas, and prostate tissues in both HD and LD animals. AUC_0–24 h_ and AUC_0‑∞_ were similar for both doses (Tables S1 and S2). The serum AUC_0–24 h_ was approximately 10-fold higher in the HD compared to the LD group, suggesting higher exposure of PCB28 to the systemic circulation following the pulmonary exposure. The dose-proportional increase in the serum AUC profile upon increasing the i.t. PCB28 dose suggests linear toxicokinetic behavior after its pulmonary uptake possibly due to avoidance of hepatic first-pass metabolism, which would occur following oral delivery. In contrast, supra-dose-proportional increases were recorded in adipose tissue, skin, muscle, spleen, and prostate from the HD animals with 18.7-, 15.1-, 15.7-, 16.4-, and 17.1-fold higher AUC compared to the LD, respectively (Figure 3B–D, Tables S1 and S2). This suggests nonlinear toxicokinetic behavior of PCB28 in these tissues. Compared to the LD rats, the *T*_1/2_ in HD rats was generally longer, with the highest *T*_1/2_ of 58 h in the skin, and indeterminate in adipose, demonstrating that longer postexposure sampling is required to determine the terminal elimination rate and subsequent *T*_1/2_ for the adipose and other fat-containing tissues. The *T*_1/2_ in the lungs is comparable to *T*_1/2_ in the brain, liver, heart, muscle, and testis.

Another important distributive toxicokinetic parameter is the xenobiotic uptake into blood cells and partition coefficient.^80,81^ The partition coefficients estimated using NCA based on the ratios of AUC from zero to infinity in blood to the AUC in the respective tissues Pt (AUC_0‑∞_tissue_/ UC_0‑∞_blood_) after lung dosing are presented in Table 2.

**Table 2:** Mean Partition Coefficients (Pt) Estimated from the Ratios of Extrapolated AUC_0‑∞_ in Blood to AUC_0‑∞_ in the Respective Tissues after Lung Dosing of [^14^C]-PCB28^a^

	high dose (HD)	low dose (LD)
tissues	Pt (AUC_0‑∞_tissue_/AUC_0‑∞_blood_)	Pt (AUC_0‑∞_tissue_ AUC_0‑∞_blood_)
blood/plasma ratio	0.89	0.89
lung	8.99	7.22
liver	5.47	6.09
kidney	5.01	4.76
adipose	35.5	19.9
skin	53.4	11.2
muscle	2.39	1.70
heart	3.44	3.24
brain	2.56	2.46
testis	1.53	1.24
spleen	3.41	1.77
bladder	2.85	3.50
prostate	25.9	7.48
bronchi	45.8	35.2
trachea	26.0	52.6
esophagus	20.7	94.5
thyroid	13.0	25.8
pancreas	20.9	8.22
thymus	3.56	1.77

a AUC_0‑∞_, area under the concentration–time curve from zero up to ∞ (AUC_0‑∞_ = AUC_0–24_ + C_last_/Ke) using known observed concentration and estimated half-life values.

We observed a blood-to-serum ratio of less than unity (i.e., 0.89), suggesting that red blood cells and clotting factors did not sequester PCB28 (Table 2). The serum-to-tissue ratios of greater than unity in brain, liver, adipose, and other distributive compartments show extensive distribution of PCB28 following lung exposure.

### 3.5 Concentration–Time Profile and Elimination of [^14^C]-PCB28

Several tissues from both doses exhibited a biphasic decline, presumably with subsequent redistribution of radioactivity (Figures 3A–H and 4A–F). After HD exposure between 12 and 200 min, most tissues (e.g., trachea, lung, thyroid, salivary gland, blood, and heart) experienced a dramatic reduction in concentration, followed by a slower elimination phase (Figure 3A,C). Radioactivity followed a decreasing trend in the lung, blood, liver, and kidneys after 12 min, followed by an upward trend in the skin, muscle, and adipose tissue (Figure 3A,B). Skin and adipose tissue concentrations peaked at 100 and 200 min, respectively, and remained at an elevated level throughout the time course (Figure 3B). In contrast to the LD exposure, skin and adipose tissue concentrations continued to increase after 200 min, peaking at 720 min postexposure (Figure 4B). Throughout the entire time course, adipose [^14^C] concentration increased 21-fold in the LD and 6.5-fold in the HD groups. Despite the observed supra-dose-proportional increase in bioaccumulation of PCB28, adipose tissue contributed 28% to total radioactivity at the LD, and 30% at the HD by 720 min postexposure (Figure 2). Several other tissues either showed an increased concentration or displayed slow to no elimination (Figures 3B–D and 4A–D). For the GI tract, the concentration of ^14^C-PCB in the intestines, digestive matter, and excreta remained elevated, apart from the duodenum digestive matter, which reached a peak at 50 min in both doses (Figures 3E,F and 4E,F).

Similar trends were observed for the [^14^C] concentration in the small intestines, digestive matter contents in the colon, and rectal ampulla for both doses. While limited, the concentration of [^14^C]-PCB28 in urine declined rapidly after the peak at 12 min exposure, for both doses, suggesting rapid excretion of the more polar metabolites or further metabolism to a relatively lipophilic methyl sulfone metabolite (Figure 3G,H). In contrast, the fecal concentration peaked at the end of the time course for both doses.

### 3.6 Metabolism and Metabolites

PCB28 and its metabolites were analyzed in samples from serum, liver, adipose tissue, and kidney for both doses. Our findings revealed that parent PCB28 dominated the quantified moieties across all time points for all of the analyzed tissues. The metabolic profile in serum demonstrates an overwhelming dominance of parent compound, which reached a peak at 12 min (7.6 dpm/mg for the LD and 47 dpm/mg for the HD), with only small contributions from conjugated metabolites (Figures 5A1,A2 and 6A1,A2). Similarly, the parent compound dominated in the liver in both HD (Figure 5B1,B2) and LD (Figure 6B1,B2). Both doses exhibited an initial fast declining phase with a subsequent slower phase, suggesting a fast initial metabolism and subsequent slow metabolism or recirculation to the liver. For the HD exposure, putative glucuronides and sulfated, and MeSO_2_ metabolites (phase II metabolites) displayed a second peak on the serum and liver concentration–time curves at 25 to 50 min after exposure, with a subsequent decline to minimal concentrations (Figure 5A1,B). The second peak was not observed in the same tissues following LD exposure. The concentrations of metabolites quantified in adipose tissue were not substantial in either dose group (Figures 5C1,C2 and 6C1,C2). Although the concentrations of hydroxylated, methyl sulfone, conjugated, and unextractable metabolites were similar in both LD and HD profiles, the disposition of parent PCB28 showed dose-dependent behavior in adipose tissue: in the LD, the concentrations increased by 3-fold between 200 and 720 min time points compared to the plateauing in the HD profile.

**Figure 5:**
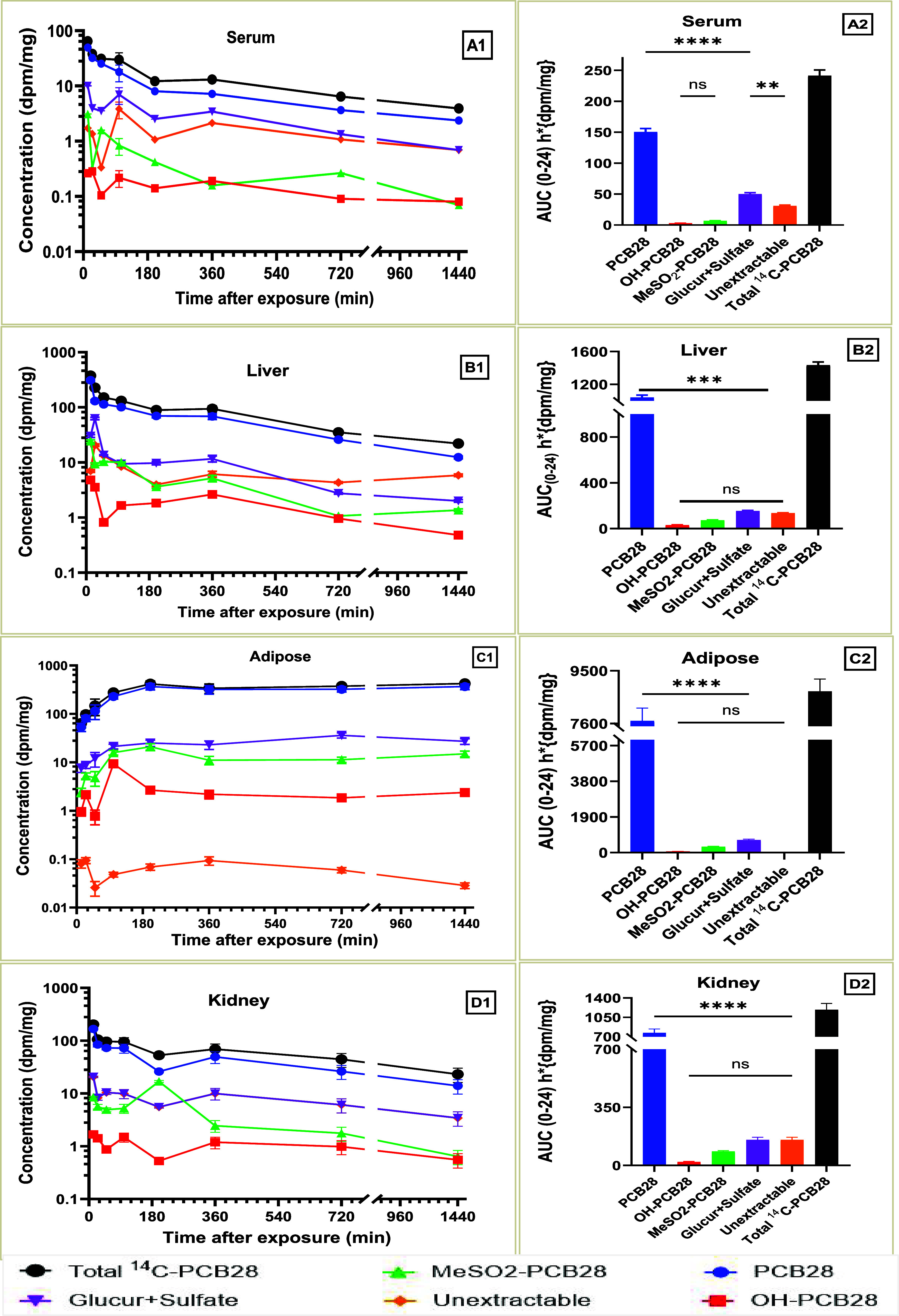
Time course of [^14^C] concentration change in fractions representing parent PCB28 and its metabolites in the serum (A1, A2), liver (B1, B2), adipose tissue (C1, C2), and kidney (D1, D2) after intratracheal instillation of a high dose [^14^C]-PCB28. ns *P* > 0.05, ** *P* ≤ 0.01, *** *P* ≤ 0.001, **** *P* ≤ 0.0001 (Kruskal–Wallis test).

**Figure 6:**
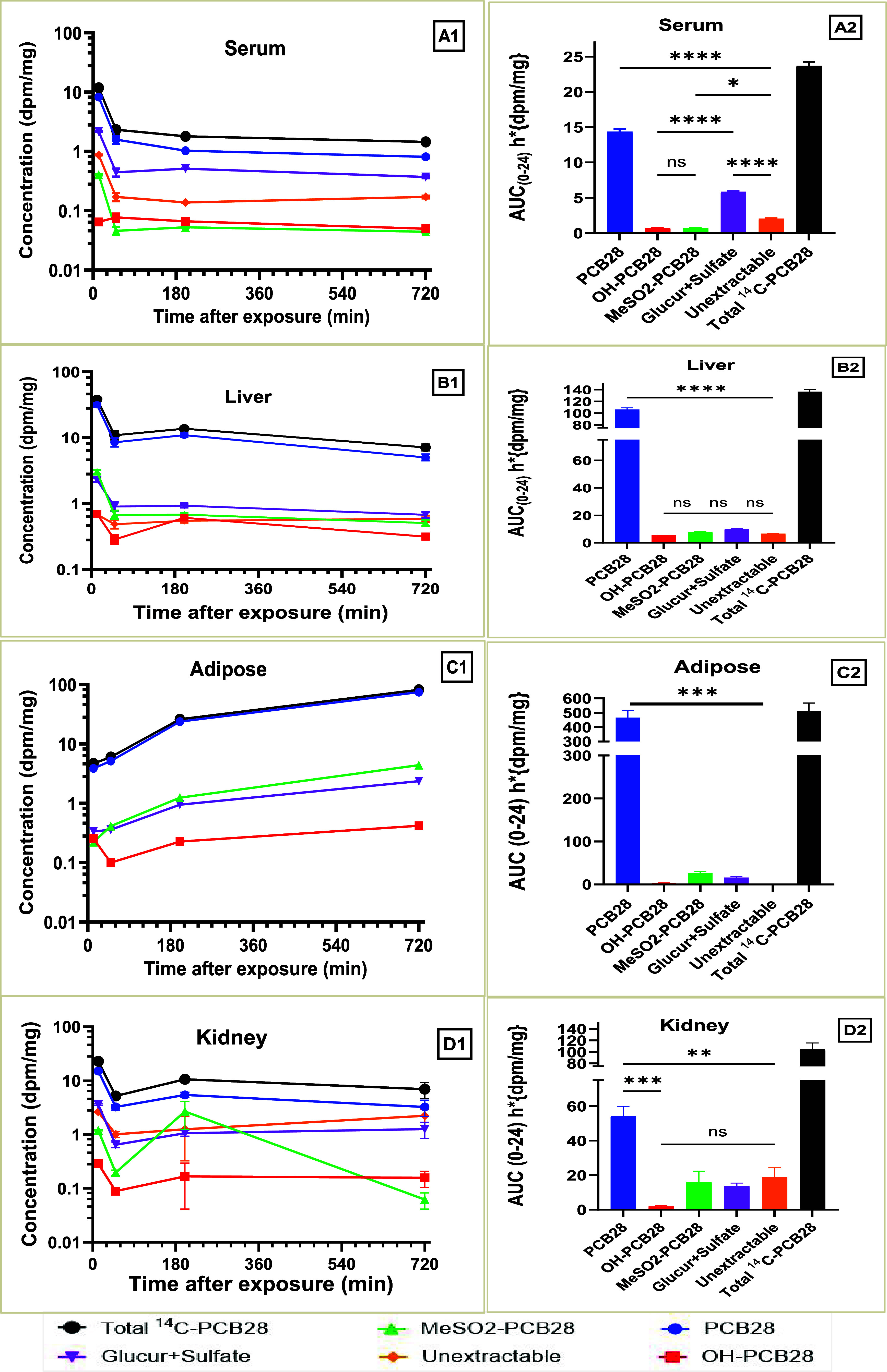
Time course of [^14^C]-PCB28 concentration change in fractions representing parent PCB28 and its metabolites in serum (A1, A2), liver (B1, B2), adipose (C1, C2), and kidney (D1, D2) after intratracheal instillation of a low-dose. Ns, *P* > 0.05, * *P* ≤ 0.05, ** *P* ≤ 0.01, *** *P* ≤ 0.001, **** *P* ≤ 0.0001 (Kruskal–Wallis test).

The final LD concentration was nearly double the expected level relative to the HD at 720 min (Figures 5C1,C2 and 6C1,C2), reaffirming our observed nonlinear kinetic profile of PCB28 in the adipose tissue compartment. Looking at the entire study time course, LD parent PCB28 concentrations increased by 36-fold compared with the 9-fold increase in the HD concentration profile. The concentration–time profile of the conjugated metabolites in the kidney was similar to the profile in the liver, except the second peak concentrations appeared sooner in the liver compared to the kidney for both doses (Figure 5B1,B2 and D1,D2). MeSO_2_ metabolites from the HD liver samples remained low, except at 200 min when they reached a peak of 21 dpm/mg (Figure 5D1,D2).

In both doses, putative OH-PCB28 levels remained low throughout the time course, never contributing more than 6% to the total radioactivity in each analyzed tissue (Figures 5 and 6). The exposure levels of individual metabolites expressed by their respective AUC_0‑∞_ are shown in Figures 5A2–D2 and 6A2–D2. The AUC_0‑∞_ of parent PCB28 was significantly higher than the AUC_0‑∞_ of the individual metabolites, suggesting limited PCB28 metabolism and extensive exposure to parent PCB28 over its metabolites at 24 h after exposure. However, the AUC_0‑∞_ profiles between metabolites were not significantly different, except for the AUC_0‑∞_ of glucuronides and sulfated metabolites in the serum, which was significantly higher than the AUC_0‑∞_ of the other serum metabolites (Figure 5A2).

## 4 Discussion

As inhalation is increasingly the major route of exposure for LC-PCBs, quantifying the time-course disposition of PCB28 from lung dosing is important for accurate exposure characterization and hazard assessment. The objective of this study was to determine fundamental toxicokinetic profiles of an environmentally relevant trichlorobiphenyl, PCB28, by elucidating its biological fate to support risk assessment. The whole-body biodistribution data presented in this study provide new insights into the absorption and disposition of PCB28 into tissues following HD and LD lung delivery. The new experimental blood-to-serum ratio of PCB28 is less than unity (i.e., 0.89) at both doses, suggesting that red blood cells and clotting factors did not sequester PCB28. The blood-to-plasma ratios are essential distributive parameters for determining the affinity of xenobiotics for erythrocytes or their presence completely in the plasma compartment. This parameter supplements the physicochemical properties, such as molecular weight or LogP, for *in vitro* prediction of toxicokinetic parameters.^82,83^ One epidemiological study reported a greater than unity experimental blood-to-plasma ratios of the HC-PCB congeners.^84^ PCBs are highly lipophilic compounds, with high plasma protein binding.^85–87^ Moreover, the partitioning results presented here demonstrate that the rate of distribution of PCB28 to erythrocytes is slow relative to the distribution rate to other tissue compartments. The dose-dependent variation in the partition coefficients (Pt) observed in this study highlights important mechanistic differences in the tissue distribution of PCB28. In the high-dose group, Pt values were highest in adipose tissue, skin, and bronchi, consistent with the saturation of plasma protein binding sites and enhanced partitioning into lipid-rich and highly perfused compartments. In contrast, the low-dose group exhibited the highest Pt values in the esophagus and trachea, suggesting that at lower concentrations, PCB28 preferentially accumulates in tissues with direct exposure and mucosal affinity, where binding dynamics remain unsaturated. These findings highlight the influence of dose on distribution kinetics and tissue-specific retention and suggest that the saturation of systemic binding and clearance mechanisms may shift the distribution profile. This has implications for PBPK modeling and risk assessment, particularly in scenarios involving chronic low-dose versus acute high-dose inhalation exposures.

Our results showed that [^14^C]-PCB28 follows linear toxicokinetic behavior, which resulted in a dose-proportional increase in the area under the serum concentration–time curve. Previous toxicokinetic studies of PCBs have predominantly focused on oral and intravenous exposure.^29,88^ In contrast, this study provides novel insights into the behavior of human-relevant LC-PCBs, PCB28, following intratracheal instillation, revealing rapid pulmonary absorption, rapid systemic distribution, and dose-dependent partitioning into peripheral tissues. Specifically, this study provides additional PCB lung absorption efficiency data required for the determination of pulmonary absorption rates.^19^ The observation of near-complete PCB28 lung uptake is comparable to the pulmonary uptake previously reported for LC-PCBs.^21–23^ While pulmonary uptake has been reported for other LC-PCBs,^21^ this study is the first to comprehensively characterize the toxicokinetics of inhaled [^14^C]-PCB28 using intratracheal instillation, with detailed time-course data across 36 tissues and multiple biological compartments. The novelty of this study lies in the integration of ADME data at both low and high doses, enabling dose-dependent comparisons of the partitioning behavior, clearance kinetics, and tissue retention.

Regarding metabolism, our findings show that PCB28 undergoes limited biotransformation in vivo, with the parent compound dominating across liver, kidney, serum, and adipose tissue. Our predicted metabolites largely align with experimental evidence for major mono–OH metabolites and sulfation pathways. Several predicted hydroxylated metabolites of PCB28, such as 2′-OH-PCB28, 3-OH-PCB28, 3′-OH-PCB28, 4′-OH-PCB31, 4-OH-PCB25, 5-OH-PCB28, and 5'-OH-PCB28, have been identified in vivo in both human plasma, rat and mouse tissues and flies following PCB28 exposure.^29,36,42,45,69–74^ The 3-sulfate-PCB28, 4-sulfate-PCB25, and methyl sulfone metabolites have been experimentally detected,^42,73,75–77^ while di–OH, tri–OH, dechlorinated, glucuronides, and sulfates metabolites have been detected or indirectly inferred, but not experimentally confirmed.^71,76,89^ Several analytical techniques were employed in the confirmation and detection of those metabolites, including liquid chromatography–tandem mass spectrometry (LC-MS/MS) and gas chromatography–mass spectrometry (GC-MS), with synthetic standards aiding structural validation in rat, mouse, fly, and human hepatic microsomes; recombinant SULT assays; and LSC for radiolabeled kinetic studies. Notably, CYP1A2 and CYP2A6 were experimentally validated as key enzymes mediating the formation of para- and meta-hydroxylated metabolites.^71^ These results align with prior literature on PCB28 and similar congeners, reinforcing the relevance of CYP-mediated biotransformation pathways and supporting the predictive accuracy of computational models when integrated with empirical data.

In this study, conjugated metabolites were primarily detected in urine, suggesting rapid conversion of phase I metabolic products to phase II metabolites and subsequent excretion, while hydroxylated and MeSO_2_–PCB metabolites remained minor. This limited phase I metabolism is consistent with PCB28’s substitution pattern, which lacks vicinal nonchlorinated positions in the meta- and para- positions, reducing susceptibility to CYP-mediated hydroxylation. Subsequently, this study identified native PCB28 in serum as a reliable biomarker of exposure, given its dominance in systemic circulation and limited metabolism. This supports its use in biomonitoring programs, particularly for vulnerable populations, such as children and school staff exposed to PCB-contaminated indoor air.

This study provides key toxicokinetic parameters that are essential for exposure characterization and hazard assessment. These data support the development of PBPK models that inform reference exposure level (REL) establishment.^90^ The study’s relevance is heightened by the environmental ubiquity of PCB28 in indoor air, especially in schools, and its potential health impact on vulnerable populations.

The disposition profiles of PCB28 observed in this study were similar to those seen in earlier studies of other congeners in rodents following intravenous and oral administrations, in that LC-PCBs concentrations in liver and muscle peaked within 15 min of dosing.^42,91^ Our study demonstrated that absorption efficiency following intratracheal exposure is much higher than the absorption rates previously reported after single-dose oral exposures to technical PCB mixture, where serum concentrations did not peak until 2 to 12 h postexposure.^19^ Furthermore, pulmonary exposure demonstrates faster attainment of peak adipose tissue concentration (3.3 h) of PCB28 compared to the previously reported peak adipose concentration of PCB28 at 24 h after oral exposure to a PCB mixture.^41^ These contrasting findings with oral exposure studies are due to PCB28 undergoing hepatic metabolism, leading to the formation of hydroxylated metabolites with distinct elimination kinetics and tissue retention profiles.^74,76^ Moreover, oral exposure results in significant hepatic induction of cytochrome P450 enzymes and metabolite formation, while pulmonary exposure appears to bypass early hepatic metabolism, allowing greater retention of the parent compound in peripheral tissues.^18,29,69,76,92^ These route-specific differences illustrate the importance of considering exposure pathways when characterizing the ADME of PCB congeners. They also highlight the need for route-specific data to inform PBPK modeling, hazard, and risk assessment frameworks.

Additionally, our findings highlight the influence of the PCB mixture versus individual congeners on PCB toxicokinetic profiles. The metabolism of certain congeners in PCB mixtures is inhibited by other congeners in the mixture, and nonmetabolized congeners can inhibit the metabolism of another.^93,94^ An increasing body of evidence from in vitro HEK293-CYP2A6 cells and in vivo animal and human studies demonstrate that individually administered PCB28 undergoes metabolism primarily via CYP2A6, producing complex mixture of metabolites, including hydroxylated metabolites, leading to cytotoxic and cytostatic effects through reactive intermediates.^37,70,71,95,96^ However, in Aroclor mixtures such as Aroclor 1016, 1242, or 1248, the metabolic behavior of PCB28 can be altered due to competitive inhibition, enzyme induction, or synergistic effects with other PCB congeners, affecting its bioavailability and toxicity.^97^ This complexity is often overlooked in risk assessments that rely solely on Aroclor-based toxicity data. The importance of individual PCB congener risk assessment is even more critical for volatile, lower-chlorinated PCBs like PCB28. The environmental degradation and weathering effects change the Aroclor mixture congener composition over time, making Aroclor-based toxicity data less representative of actual exposures.

The results of the initial disposition of [^14^C]-PCB28 to the highly perfused tissues and subsequent redistribution to adipose and skin were comparable to previous findings in rats exposed to a similar dose of [^14^C]-PCB11, where the [^14^C] activity distributed to muscle and liver tissue as early as 12 min postexposure.^21^ Similar tissue disposition profiles were reported following exposure to PCB52 in rats and mice.^22,46,47,69^ Furthermore, this profile is consistent with findings from the disposition and toxicokinetics studies of persistent organic pollutants in many animal species, where initial uptake in the liver and muscle is commonly found, evidently due to the high blood perfusion rate to the liver and the substantial proportion of body weight of the muscle.^22,47,69,98,99^ The comparatively faster distribution of the native PCB28 in the HD could be due to the saturation of binding sites in serum by OH-PCBs and sulfated PCB conjugates which have a high affinity for binding to serum proteins.^29,87^ At HD, these binding sites may have become saturated, leaving the parent compound and MeSO_2_–PCB28 metabolites available for direct distribution to the skin and adipose tissues. Furthermore, the slower distribution of PCB28 in the LD may be attributed to the high plasma protein binding of PCBs and potential futile cycling behavior of their metabolites.^87,100–103^ This futile cycling behavior could increase downstream cellular reactive oxygen species level, resulting in neurotoxicity.^102,104,105^

The estimated *T*_1/2_ for PCB28 following intratracheal exposure in this study is comparable to the *T*_1/2_ of inhaled PCB11 at comparable doses.^21^ In our prior study, we found that the serum PCB11 elimination half-life was 11.5 h after acute inhalation exposure to a PCB mixture at an estimated dose of 40 μg/rat, which was comparable to the estimated values following HD exposure in this study. In contrast, other previous studies following intravenous and oral exposure to other PCB congeners reported relatively longer half-lives, ranging from 1 to 2 days.^40,49^ In agreement with the previous studies, the disappearance of PCB28 from major organs in this study follows biphasic first-order kinetic pattern.^40,49^ Biphasic behavior has been widely found for higher-chlorinated congeners in rats exposed to PCBs both orally and intravenously.^39,42,106^ The biphasic decline in PCB28 concentrations observed across various tissues reflects distinct physiological processes governing its distribution and clearance. The initial rapid decline between 12 and 200 min in tissues such as the trachea, lung, thyroid, salivary glands, blood, and heart likely represents redistribution from highly perfused organs into lipid-rich compartments. This phase may be influenced by rapid plasma-tissue exchange and initial binding to plasma proteins, such as albumin and lipoproteins, which are known carriers of PCBs. At higher doses, the saturation of these binding sites increases the free fraction of PCB28, accelerating its movement into peripheral tissues. The second, slower phase of elimination is consistent with redistribution into adipose tissue, skin, and muscle, where PCB28 accumulates due to its high lipophilicity and limited local metabolism. These compartments function as deep depots with slow back-diffusion and prolonged retention. Concurrently, hepatic biotransformation and biliary excretion, both capacity-limited processes, may become saturated, further extending systemic exposure and reinforcing the nonlinear toxicokinetics observed at high doses.

While our findings on elimination profiles are consistent with prior observations that the elimination rate of LC-PCB congeners increases with decreasing degree of chlorination and availability of unsubstituted carbons at para-/meta-positions, it is important to note that elimination kinetics are influenced not only by chlorination degree and substitution patterns but also by exposure route, dose level, and compound-specific properties. This study investigated a single congener (PCB28) via intratracheal instillation, and thus, broader generalizations should be made cautiously. The observed biphasic elimination and dose-dependent distribution patterns underscore the complexity of PCB toxicokinetics and highlight the need for congener-specific and exposure-contextual data to inform risk assessment and PBPK modeling.

The supra-dose-proportional increased [^14^C] concentration in adipose tissue after exposure to the LD may be attributed to several factors. First, PCB congeners may undergo metabolism to form neutral MeSO_2_–PCB metabolites with a slightly lower lipophilicity than the parent PCB28 via a multistep pathway involving glutathione conjugation and mercapturic acid pathway, resulting in a sustained circulating level of MeSO_2_ metabolites together with the parent PCBs. These recirculating metabolites are potentially more toxic than the parent compounds.^107,108^ Second, dose-dependent distribution of PCBs was observed in a subacute oral exposure study in rodents, where several dioxin-like congeners increased in concentration in adipose tissue, as the dose increased.^109^ The supra-dose-proportional increases in PCB28 AUC observed in adipose tissue, skin, and other lipid-rich compartments suggest a dual mechanism involving both enhanced partitioning and saturation of elimination pathways. PCB28, like other lipophilic congeners, exhibits a strong affinity for plasma proteins, particularly albumin and lipoproteins, which act as carriers in systemic circulation. However, at high doses, these binding sites may become saturated, increasing the free fraction of PCB28 and facilitating deeper partitioning into lipid-rich tissues, such as adipose and skin. This is supported by theoretical models showing that serum lipid content significantly influences PCB partitioning between serum and adipose tissue.^110^ Concurrently, hepatic biotransformation, primarily via CYP2A6, and biliary excretion may reach capacity limits, reducing clearance efficiency and prolonging systemic exposure.^71^ Together, these processes likely contribute to the observed nonlinear toxicokinetics, underscoring the importance of dose-dependent saturation phenomena in the disposition of PCB28.

The concentration gradients of the [^14^C]-PCB28 radioactivity in the GI tract matter were expected to be dictated by the gastrointestinal motility and environmental condition until its eventual excretion via feces. However, our findings suggested lower levels of activity in the feces than in the remaining GI segments throughout the time course in both doses. This observation suggested two explanations: (1) PCB28 underwent passive excretion into the GI tract from the systemic circulation following pulmonary absorption and subsequent redistribution. (2) The excretion of chlorinated compounds, including PCBs from systemic circulation to intestinal lumen via both biliary and nonbiliary routes, has been previously reported.^111,112^ Moreover, microbiome-aided transformation of parent PCB to metabolites and subsequent reabsorption is an additional postulate. The effects of microbiome on the disposition of PCBs have been reported.^45,113^ Also, the high octanol–water partition coefficient of PCBs makes them potential molecules to readily undergo GI transport through diffusing across cell membranes. The available evidence demonstrated high absorption efficiency of PCB28 following oral administration,^114,115^ suggesting high uptake efficiency across the GI wall and subsequent redistribution, and variable [^14^C] concentration in many tissues throughout the time course. For example, immediately following the peak concentration of PCB28 in stomach matter radioactivity at 50 min, the levels in several tissues were elevated between 50 and 100 min, indicating an apparent redistribution of the radioactivity from the stomach to those elevated tissues. The high lipophilic property of PCBs may make them good candidates for GI tract absorption via lymphatic circulation like other lipid-soluble compounds, thus avoiding first-pass metabolism in the liver.^114^ An additional explanation for the fluctuations in the concentrations in the GI tract contents is the documented roles of enterohepatic cycling in the intestinal deconjugation of conjugated metabolites and subsequent reabsorption of the hydroxylated metabolites into the systemic circulation.^116^

Urinary [^14^C] concentration peaked at 200 min in both doses and consisted solely of conjugated metabolites, suggesting that transformation from phase I to phase II occurred rapidly and more polar metabolites were readily excreted in the urine or further transformed to more stable metabolites contributing to the recirculating compounds. The parent PCB28 was initially oxidized by CYP enzymes to arene oxide intermediates, preferentially in the meta–para position, followed by 1,2 migration reaction (NIH shift) as shown in Figure 1 and suggested elsewhere.^29,117^ Alternatively, it could also undergo direct insertion of hydroxyl groups, leading to the metabolites 3-OH-PCB28 and 3′-OH-PCB28, forming toxic hydroxylated metabolites.^29,102^ The lower levels of OH-PCB metabolites in this study may be attributed to the further conjugation of the unstable OH-PCBs by uridine diphosphate glucose (UDPG) or sulfotransferases (SULT) enzymes to their respective glucuronide and sulfate metabolites, serving as precursors to the formation of conjugated metabolites,^29,72,102^ particularly in the liver or kidneys.^118^ A finding from Dhakal et al. suggested that LC-PCBs are largely metabolized via sulfation.^74,76^ PCB28 underwent limited metabolism following lung dosing. The limited PCB28 metabolism in this study was presumably due to the use of the intratracheal administration route that bypasses the hepatic first-pass effect, where sulfation could occur. As highlighted by Duffel and Lehmler (2024), hydroxylated PCB metabolites (OH-PCBs) of LC-PCBs are extensively conjugated by cytosolic sulfotransferases (SULTs) to generate PCB sulfates.^76^ The additional plausible contributor to the limited PCB28 metabolism was the absence of neighboring nonsubstituted carbons in the meta- and para-positions; a substitution pattern that increases the chances of CYP-mediated biotransformation, resulting in more polar metabolites and urinary excretion. The retention of parent PCB28 in the body suggests the potential of using the parent compound in the serum as a better biomarker for acute exposure to PCB28 than conjugated metabolites in the urine. In contrast, PCB11 was extensively metabolized primarily into sulfated and glucuronide conjugates and excreted via urine and feces due to the presence of nonsubstituted carbons in the meta- and para-positions.^21^ The propensity for urinary excretion is common in lower-chlorinated congeners, which is propelled by a decrease in chlorination and increased solubility.

The administered low dose in this study is higher than typical environmental concentrations but close to what a person would attain living in a high concentration residential environment (e.g., indoor air levels reaching 1689 ng/m^3^).^11,31,119^ An adult person breathing this concentration of PCBs would be expected to attain an inhaled dose of 27 μg/day or 0.4 μg/kg-day. The low dose used in this study (16.4 μg/kg-day) represents about 40 days of high residential exposure. The two selected doses (42 μg/rat and 4.2 μg/rat) reflect a range of exposures that allow for the detection of tissue-specific toxicokinetic patterns and potential dose-dependent metabolic saturation.^120,121^ The two doses were selected to align with prior inhalation exposure estimates for PCB11 and to ensure sufficient radiolabeled compound for detection across tissues.^21,30,61^ The tissue-specific kinetic data will facilitate the extrapolation of tissue concentrations to environmentally relevant exposures.

These overall findings have important implications for evaluating human exposure, particularly in school environments. The U.S. Environmental Protection Agency (EPA) has established Exposure Levels for Evaluation (ELEs) for PCBs in indoor school air to ensure that total daily exposure remains below the oral reference dose (RfD) of 20 ng/kg-day. ELEs range from 100 ng/m^3^ for toddlers to 600 ng/m^3^ for adolescents, with 500 ng/m^3^ recommended for adults. These benchmarks assume background exposure from diet and dust and are established to be health-protective, especially for vulnerable populations. Our results suggest that even low-level inhalation exposures can result in systemic distribution and tissue accumulation of PCB28, with limited clearance over time. The minimal metabolism and low urinary excretion support the use of serum PCB28 as a reliable biomarker for inhalation exposure. Furthermore, the similarity in distribution kinetics between PCB28 and PCB11, coupled with the reduced metabolism of PCB28, highlights the need for compound-specific toxicokinetic profiles in risk modeling.

The current regulatory frameworks for PCBs, including ELEs and cleanup criteria, are predominantly based on commercial mixtures, such as Aroclors. These mixtures, defined by their chlorine content (e.g., Aroclor 1242 and 1254), were historically used in industrial applications and serve as the basis for many analytical and risk assessment protocols. However, this approach fails to capture the complexity of real-world exposures, which involve weathered, degraded, and biologically transformed PCB congeners.^36,69,95,122,123^ Environmental and biological processes, such as microbial degradation, metabolism, and selective bioaccumulation, alter congener profiles, resulting in mixtures that differ significantly from original Aroclor formulations. Moreover, individual congeners vary in their persistence, bioaccumulation potential, and toxicity. These discrepancies undermine the relevance of Aroclor-based reference values for contemporary exposure scenarios. Risk assessment and biomonitoring must focus on congener-specific and mixture-resolved approaches that reflect actual environmental and biological profiles. This includes integrating toxicokinetic data, metabolite characterization, and multiomics analyses to better assess health risks, particularly for vulnerable populations such as children and pregnant women. Congener-specific data provide more accurate estimates of exposure and toxicity, particularly for compounds such as PCB28 that may not be well represented in commercial mixtures. Our findings reinforce this approach, demonstrating that PCB28 exhibits distinct toxicokinetic behavior that warrants individual consideration in exposure assessments. Considering these findings, monitoring indoor air concentrations in schools and other public buildings remains essential. Future studies should explore chronic exposure scenarios, age-dependent and sex-dependent kinetics, and the implications of coexposure to multiple PCB congeners.

Our study had some limitations. Our findings on the levels of PCB28 metabolites should be considered with caution because the identification of all individual hydroxylated metabolites was infeasible due to a lack of analytical standards for all of the metabolites. However, given that PCB28 is not extensively metabolized in this study, as indicated by the overwhelming dominance of parent compounds found in the liver, kidney, adipose, and serum, the uncertainty in the levels of metabolites may be inconsequential. Unlike PCB11 however, less than 4% of the administered [^14^C]-PCB28 was excreted via urine and feces, resulting in 92% retention in the body or apparent bioavailability using fecal excretion.^63^

Potential losses of radioactivity were possible due to variability in the proportion of body weight represented by blood, adipose, muscle, skin, and bone and the potential error in the calculation of lymph nodes and bone marrow weights. However, the use of an intratracheal instillation approach for dosing and comprehensive mass-balance recovery of administered doses from exhaled breath, wipes, various tissues, and GI tract contents represents a robust mechanism to account for potential loss as opposed to inhalation exposure. Our whole-body disposition data would be more robust with a larger number of animal replicates. However, this was precluded by the exceedingly high cost of custom-synthesized, high-purity radiolabeled PCB28. This limitation may not have affected our study findings as indicated by the small variances in tissue concentrations from triplicate rats necropsied at 200 min in our [^14^C]-PCB11 study.^21^ In addition, the whole-body tissue sample collection at several earlier time points after lung dosing and utilizing the isotope labeling technique yields much needed time-course deposition and clearance data after pulmonary exposure. The risk of pseudo-oral exposure associated with intratracheal instillation is another potential limitation of our study. However, the consistent [^14^C] levels in the trachea, lung, and stomach tissue, following similar trends over time, reflect the reproducibility of our findings. These observations provide assurance that the slightly higher esophageal concentrations were predominantly from mucociliary clearance originating in the trachea, followed by subsequent swallowing of [^14^C]-PCB28. Also, the use of intratracheal lung dosing and comprehensive mass-balance recovery of administered doses offers a robust mechanism to avoid pseudo-oral exposure, further upsets this limitation.

Another important limitation to note is that this study exclusively evaluated male rats, limiting the results extrapolation to infer potential sex-specific TK differences that may exist in females. Future studies incorporating both sexes are warranted to fully characterize the influence of sex on the metabolism and whole-body distribution of PCB congeners, including PCB28, particularly in the context of individual versus mixture exposures.

Our study provides detailed toxicokinetic data for PCB28 following single-dose pulmonary exposure. Real-world exposures, particularly in indoor environments such as schools and homes, involve complex mixtures of PCB congeners and not isolated compounds. These mixtures may include both legacy and contemporary congeners, each with distinct physicochemical properties, metabolic profiles, and toxicological mechanisms.

Overall, this study presents new data on the tissue disposition and biological fate of [^14^C]-PCB28 after lung exposure, yielding critical insights across uptake, distribution, and elimination dynamics. Within a remarkably short period (12 min postdosing), radioactivity was distributed across 36 distinct tissue types and five compartments within the GI tract. Initial distribution favored muscle and liver in HD tissues, while LD tissues exhibited accumulation in muscle, esophagus, and trachea. Subsequent redistribution occurred with preferential accumulation in skin and adipose tissue. Intratracheal administration of [^14^C]-PCB28 demonstrated linear toxicokinetics, resulting in dose-proportional increases in serum concentrations. Tissue concentration profiles exhibited biphasic patterns, characterized by an initial rapid decline followed by a slower phase, suggestive of redistribution or recirculation. PCB28 metabolism remains limited, with the parent compound prevailing in the liver, kidney, serum, and adipose tissue. Although urinary and fecal excretion were limited, fecal excretion emerged as the primary excretory pathway within the 24 h study window.

## 5 Practical and Public Health Implications

Our findings provide a critical foundation for developing PBTK models, essential for exposure characterization and risk assessment following airborne PCB28 exposure and related congeners. First, understanding the highly efficient uptake of PCB28 after inhalation with subsequent systemic distribution enables more accurate exposure assessments. Regulatory agencies can use this information to refine exposure scenarios and establish appropriate safety thresholds. Second, the linear toxicokinetics observed, along with dose-proportional increases in concentrations in the major organs, emphasize the need for precise dosimetry models. Incorporating these data into PBTK models allows the prediction of human exposure and assessment of associated health risks. Third, the identification of serum levels of parent PCB28 as a reliable biomarker for acute inhalation exposure facilitates early detection of exposure incidents and informs intervention strategies. Additionally, our findings underscore the importance of comprehensive environmental monitoring, targeted low-level exposure and risk assessment guidelines, and future research to explore mechanisms underlying concentration–time curves and redistribution dynamics.

## Supplementary Material



## Data Availability

Data will be publicly available in a public access repository (Iowa Research Online).

## Notes

Air and excreta sampling strategy during the postexposure observation period (Figure S1); schematic diagram of the extraction and separation system used to fractionate PCB28 and its potential metabolites into individual moieties in different rat tissue samples (Figure S2); toxicokinetic parameters of PCB28 obtained by noncompartmental analysis following intratracheal administration of high-dose and low dose [^14^C]-PCB28 formulations to male Sprague–Dawley (Tables S1 and S2) (PDF)
