# Supplementary material for: Whole-Body Disposition and Metabolism of [14C]-2,4,4′-Trichlorobiphenyl (PCB28) Following Lung Administration in Rats
Source: Environ Sci Technol. 2025 Oct 23;59(43):23106–24. doi: 10.1021/acs.est.5c07982 (PMC12593371; doi:10.1021/acs.est.5c07982)
Supplement: Supplementary file 1 [file es5c07982_si_001.pdf]

Supplementary Materials:

Whole-body disposition and metabolism of [^{14}C]-2,4,4'-trichlorobiphenyl (PCB28) following lung administration in rats.

Yau Adamu[a], Nicole Marie Brandon[b], Andrea Adamcakova-Dodd[b], Hui Wang[a], and Peter S. Thorne[a,b]*

[a] *Human Toxicology Program, The University of Iowa, Iowa City, Iowa 52242, United States*

[b] *Department of Occupational and Environmental Health, The University of Iowa, Iowa City, Iowa 52242, United States*

*Corresponding author:

Peter S. Thorne −Department of Occupational and Environmental Health, University of Iowa, Iowa City, Iowa 52242, United States

ORCID: 0000-0002-5045-0929; phone:(319)335-4216; email: peter-thorne@uiowa.edu

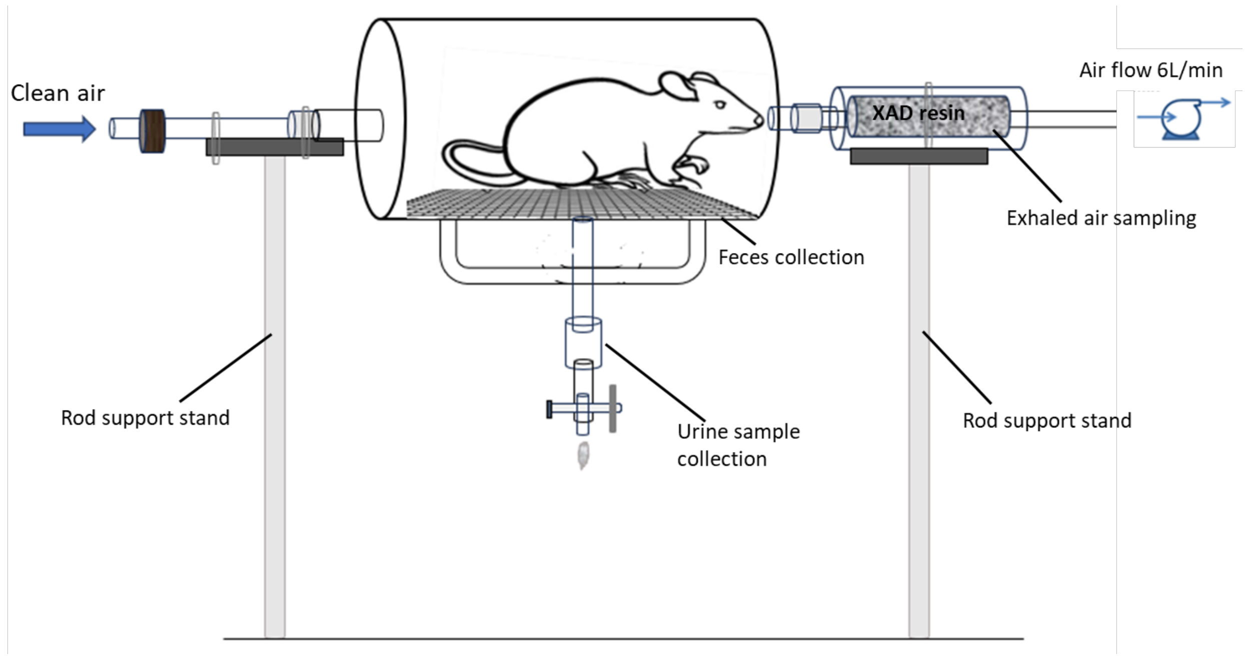

Figure S1. Schematic illustration of air and excreta sampling during the post-exposure observatory period.

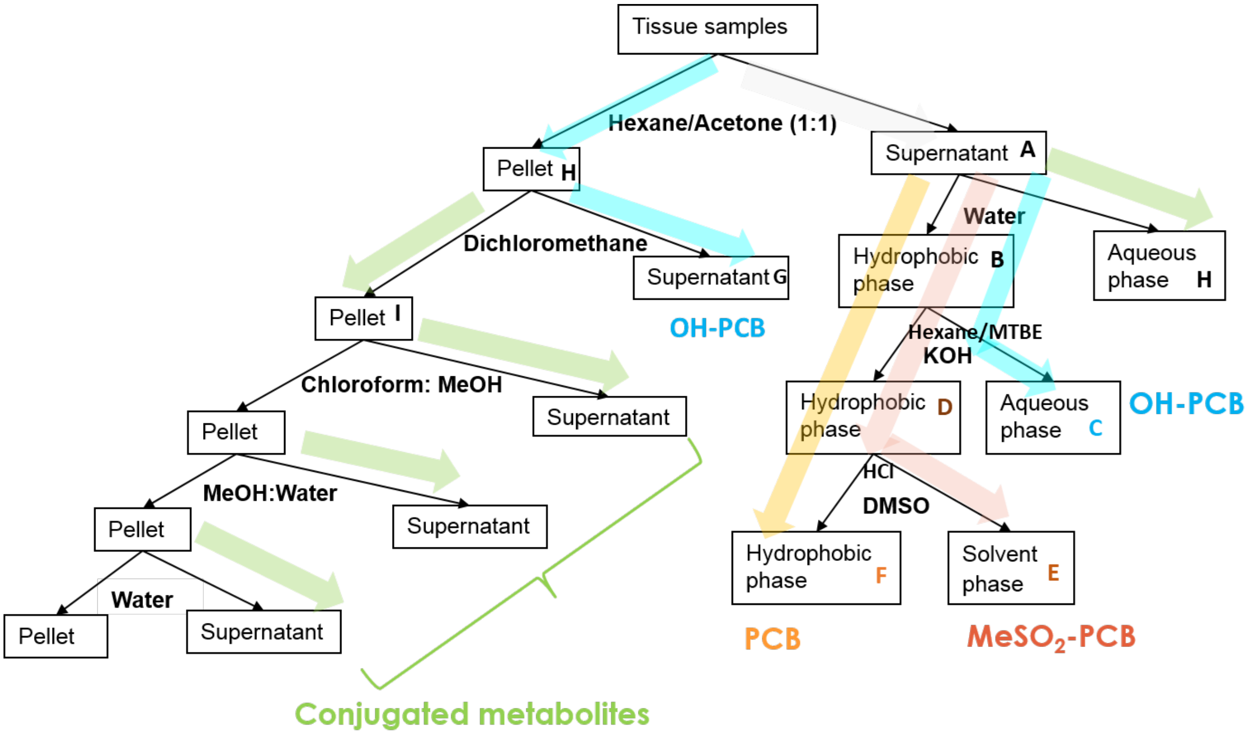

Recovery %	A	B	C	D	E	F	G	H
PCB28	100 ± 5	101 ± 4	ND	97 ± 4	10 ± 1	88 ± 3	ND	1 ± 1
4'-OH-CB25	94 ± 10	90 ± 6	105 ± 2	ND	ND	ND	16 ± 2	1 ± 0
4-OH-CB33	85 ± 18	86 ± 10	120 ± 7	ND	ND	ND	14 ± 3	1 ± 1
4-MeSO2-CB49	85 ± 11	80 ± 10	12 ± 1	84 ± 10	117 ± 1	1 ± 0	8 ± 1	ND

Figure S2. Schematic diagram illustrating the extraction and separation system for PCB28 and its potential metabolites in rat tissue samples.

Table S1. *Toxicokinetic parameters of PCB28 obtained by non-compartmental analysis after intratracheal administration of High Dose [^{14}C]-PCB28 formulations to Male Sprague Dawley rats. (Mean, n = 2–3).*

Tissue compartment	$T_{1/2}$ h	T_{max} h	C_{max} dpm/mg	C_{24} dpm /mg	$AUC_{0\text{-}24}$ h*dpm/mg	$AUC_{0\text{-}\infty}$ h*dpm/mg	Vz/F mg	Cl/F mg/h	Pt ($AUC_{0\text{-}\infty\_tissue}$ /$AUC_{0\text{-}\infty\_blood}$
Serum	11.7	0.21	65.0	4.0	243	311	5.83	0.34	1.00
Lung	10.4	0.21	603	44.0	2140	2790	0.57	0.04	9.0
Liver	8.3	0.21	376	22.0	1440	1700	0.80	0.06	5.5
Kidney	11.2	0.21	204	23.0	1190	1560	1.11	0.07	5.0
Adipose	ND	3.33	435.	385	8630	ND	ND	ND	35.5
Skin	56.8	1.67	245	155	3900	16600	0.53	0.01	53.4
Muscle	8.5	0.21	77.0	10.0	621	743	1.80	0.14	2.4
Heart	9.3	0.21	234	17.0	841	1070	1.35	0.10	3.4
Brain	8.1	0.21	206	11.0	667	796	1.57	0.13	2.6
Testis	10.4	0.21	51.0	6.0	385	476	3.38	0.23	1.5
Spleen	14.8	0.21	98.0	18.0	675	1060	2.16	0.10	3.4
Bladder	6.3	0.21	125	8.0	813	886	1.10	0.12	2.9
Prostate	39.6	3.3	177	89.0	2960	8050	0.76	0.01	25.9
Bronchi	8.1	0.21	2830	205	11800	14200	0.09	0.01	NA
Trachea	12.7	0.21	1950	86.0	6500	8070	0.24	0.01	NA
Esophagus	8.1	0.83	10100	13.0	6280	6430	0.19	0.02	NA
Thyroid	14.0	0.21	560	72.0	2580	4040	0.54	0.03	13.0
Pancreas	26.4	0.21	225	93.0	2950	6490	0.63	0.02	20.9
Thymus	15.2	0.42	85.9	23.0	602	1110	2.12	0.10	3.6

NA, not applicable; ND, not determined; $AUC_{0\text{-}\infty}$, area under the concentration-time curve from zero up to ∞ with extrapolation of the terminal phase calculated with the equation $AUC_{0\text{-}\infty} = AUC_{0\text{-}24} + C_{last}/Ke$ using known observed concentration and estimated terminal elimination half-life values. Cl/F, apparent total serum clearance of ^{14}C-PCB28 after intratracheal administration estimated from $CL/F = Dose/AUC_{0\text{-}\infty}$; Vz/F, apparent volume of distribution during terminal phase for non-steady-state after extravascular administration estimated from $Vz/F = Dose_{\_ex}/(AUC_{0\text{-}\infty} * Ke)$.

Table S2*. Toxicokinetic parameters of PCB28 obtained by non-compartmental analysis after intratracheal administration of Low Dose [^{14}C]-PCB28 formulations to Male Sprague Dawley rats. (Mean, n = 2–3).*

Tissue Compartment	$T_{1/2}$ h	T_{max} h	C_{max} dpm/mg	C_{24} dpm/mg	AUC_{0-24} h*dpm/mg	$AUC_{0-\infty}$ h*dpm/mg	Vz/F mg	Cl/F mg/h	Pt ($AUC_{0-\infty\_tissue}$/ $AUC_{0-\infty\_blood}$
Serum	6.49	0.20	9.55	0.80	23.1	30.6	3.28	0.350	1.00
Lung	5.7	0.20	62.9	6.4	167	219	0.40	0.049	7.2
Liver	7.9	0.20	30.6	5.7	141	205	0.59	0.052	6.1
Kidney	22.2	0.20	18.3	5.6	110	288	1.1	0.037	4.8
Adipose	ND	12.00	63.7	63.7	459	ND	ND	ND	19.9
Skin	ND	12.00	34.2	34.2	258	ND	ND	ND	11.2
Muscle	ND	0.20	4.0	3.2	39.4	ND	ND	ND	1.7
Heart	8.8	0.20	31.0	3.2	74.9	115	1.2	0.093	3.2
Brain	11.4	0.20	11.1	2.4	56.8	96.1	1.8	0.111	2.5
Testis	ND	3.33	3.6	1.6	28.8	ND	ND	ND	1.2
Spleen	ND	0.20	8.8	3.2	41.0	ND	ND	ND	1.8
Bladder	ND	3.33	8.4	6.4	81.0	ND	ND	ND	3.5
Prostate	ND	3.33	16.9	15.9	173	ND	ND	ND	7.5
Bronchi	8.2	0.20	167	48.6	814	1390	0.09	0.008	NA
Trachea	6.3	0.03	2900	21.5	1220	1410	0.07	0.008	NA
Esophagus	1.7	0.03	3300	8.8	2180	2210	0.01	0.005	NA
Thyroid	3.0	0.03	2180	9.6	595	637	0.07	0.017	25.8
Pancreas	ND	12.0	29.5	29.5	190	ND	ND	ND	8.2
Thymus	ND	0.03	9.7	4.8	41.0	ND	ND	ND	1.8

NA, not applicable; ND, not determinable; $AUC_{0-\infty}$, area under the concentration-time curve from zero up to ∞ with extrapolation of the terminal phase calculated with the equation $AUC_{0-\infty} = AUC_{0-24} + C_{last}/Ke$ using known observed concentration and estimated terminal elimination half-life values. Cl/F, apparent total serum clearance of ^{14}C-PCB28 after intratracheal administration estimated from CL/F =Dose/$AUC_{0-\infty}$; Vz/F, apparent volume of distribution during terminal phase for non-steady-state after extravascular administration estimated from Vz/F = $Dose_{\_ex}/(AUC_{0-\infty}*Ke)$.

Table S3. *Comprehensive list of PCB28 Metabolites experimentally confirmed versus predicted*

Metabolite Type	Metabolite Name	Structure Description	Confirmed In	Reference	Prediction
Mono-hydroxylated	3-OH-PCB28	3-hydroxy-2′,4,4′-trichlorobiphenyl	Human plasma, Drosophila, CYP assays	Idda et al., 2020; Quinete et al., 2017; Randerath et al., 2024; Moir et al., 1996; Duffel & Lehmler, 2024	Predicted
Mono-hydroxylated	3′-OH-PCB28	3′-hydroxy-4′,4,6′-trichlorobiphenyl	Human plasma, CYP assays	Quinete et al., 2017; Randerath et al., 2024; Moir et al., 1996; Duffel & Lehmler, 2024	Predicted
Mono-hydroxylated	4′-OH-PCB25	4′-hydroxy-2′,3′,4-trichlorobiphenyl	Human plasma, CYP assays	Quinete et al., 2017; Randerath et al., 2024; Moir et al., 1996; Zhang, 2021; Duffel & Lehmler, 2024	Predicted
Mono-hydroxylated	4′-OH-PCB31	4′-hydroxy-2′,4′,5-trichlorobiphenyl	Human plasma, CYP assays	Quinete et al., 2017; Randerath et al., 2024; Moir et al., 1996; Duffel & Lehmler, 2024	Predicted
Mono-hydroxylated	5-OH-PCB28	5-hydroxy-2,4,4′-trichlorobiphenyl	CYP assays, rat feces	Randerath et al., 2024; Moir et al., 1996; Duffel & Lehmler, 2024	Predicted
Mono-hydroxylated	Other mono-OH-PCBs	Ortho/meta/para-substituted	Rat feces, liver, brain	Moir et al., 1996	Predicted
Dihydroxylated	Di-OH-PCB28	Not structurally specified	Rat feces; human brain (tentative)	Moir et al., 1996; Li et al., 2022; Duffel & Lehmler, 2024	Predicted
Tri-/Tetra-OH	Unspecified congeners	Detected by Nt-LCMS in human brain	Human brain	Li et al., 2022	Not Predicted
Methylthioether	3 variants	Not structurally specified	Rat feces, liver, brain	Moir et al., 1996	Inferred via Predicted
Methylsulfone	1 variant	Para-substituted	Rat liver	Moir et al., 1996	Inferred via Predicted
Dechlorinated hydroxylated	3-OH-PCB15	3-hydroxy-4,4′-dichlorobiphenyl	CYP assays	Randerath et al., 2024; Duffel & Lehmler, 2024	Not Predicted
Dechlorinated hydroxylated	Other dechlorinated OH-PCBs	Not individually named	Rat feces	Dhakal et al., 2012; Moir et al., 1996; Duffel & Lehmler, 2024	Not Predicted
Sulfated	4′-PCB25 sulfate	Sulfated derivative of 4′-OH-PCB25	Human serum	Zhang, 2021; Duffel & Lehmler, 2024	Predicted

Sulfated	Sulfate conjugates of OH-PCBs	Not individually named	Inferred via enzymatic hydrolysis or internal standards	Parker et al., 2018; Quinete et al., 2017; Randerath et al., 2024; Haga et al., 2018; Moir et al., 1996; Dhakal et al., 2012; Duffel & Lehmler, 2024	Predicted
Glucuronidated	Glucuronide conjugates of OH-PCBs	Not individually named	Inferred via enzymatic hydrolysis	Quinete et al., 2017; Randerath et al., 2024; Haga et al., 2018; Moir et al., 1996; Duffel & Lehmler, 2024	Predicted
